# Study of pressure influence on thermal transition in spin-crossover nanomaterials

**DOI:** 10.1186/1556-276X-9-691

**Published:** 2014-12-20

**Authors:** Iurii V Gudyma, Artur Iu Maksymov, Victor V Ivashko

**Affiliations:** Department of General Physics, Chernivtsi National University, Kotsjubynskyi Str. 2, 58012 Chernivtsi, Ukraine

**Keywords:** Spin-crossover nanoparticles, Ising-like model, Breathing crystal field, Statistical fluctuations, Thermal transition

## Abstract

The thermal transition accompanied by the variation of the molecular volume in nanoparticles of spin-crossover materials has been studied on the basis of microscopic Ising-like model solved using Monte Carlo methods. For considered model, we examined the spin-crossover phenomenon with applied hydrostatic pressure and thus was shown the possibility to shift transition temperature toward its room value. The obtained results of numerical simulations are in agreement with the experimental ones.

## Background

The spin-crossover molecular magnets are the new class of coordination inorganic complexes with *d*^4^ to *d*^7^ electronic configuration of metal ion orbitals, situated in the centre of the octahedral ligand field [1–3]. These materials posses two stable states: low-spin (LS) configuration with diamagnetic properties and high-spin (HS) configuration which is showing paramagnetic behavior. The spin configuration of each state is defined by the electronic configuration of transition metal ion. The spin-crossover phenomenon consists in the existence of equilibrium point between LS and HS isomers and ability of interconversion between spin states of the compounds. We focused on iron(II) compounds with coordination number 6, due to their practical applicability as sensors, data storage, switching, display, and visualization systems. The attractiveness of practical applications of these materials lies in the possibility to design IT devices with nanosized unit cells of about 4 nm ^3^ [4]. For these compounds, the total spin number for LS and HS configurations is respectively *S*=0 and *S*=2 [1].

To understand the mechanism of interconversion between LS and HS configuration, we must consider the fine structural features of spin-crossover complexes and the way of populating the high-energy levels. The ligand field action on metal ion provokes the splitting of its d-orbitals into a subset of three orbitals *d*_*x*_,*d*_*y*_, and *d*_*z*_, which are basis of the irreducible representation of *t*_2*g*_ sublevels, and a subset of two orbitals *d*_*x*1_,*d*_*y*1_ which are basis of the irreducible representation of *e*_*g*_ sublevels. For the LS configuration, the energy difference between metastable ^5^*T*_2*g*_ and ground ^1^*A*_1*g*_ state is large enough, so the strength of electron-electron repulsion is not big enough to provoke transition. In this case according to Hund’s rule, the d-electrons will nicely pair up with antiparalel spins on the *t*_2*g*_ sublevels, and we will detect the diamagnetic low-spin ground state. The electrons on *t*_2*g*_ sublevels may get additional energy from the environment, and in this way, the electron-electron repulsion is increasing. Bringing additional energy on d-electrons, it is possible by influence of external physical fields: temperature, pressure, magnetic field, light irradiation, and other [5–7]. The very recent studies of spin-crossover nanoparticles open new research subjects in the switchable molecular magnetic materials [8–10].

In the vicinity of transition point, the energy difference between ^5^*T*_2*g*_ and ^1^*A*_1*g*_ states decreases and the equilibrium between ligand field strength and electron-electron repulsion is being reached. Now, when there are no barriers between *t*_2*g*_ and *e*_*g*_ orbitals, according to the same Hund’s rule, the d-electrons will enter uniformly on the *t*_2*g*_ and *e*_*g*_ orbitals, but in this case, the spins will not be compensated which leads to a resultant magnetic moment and paramagnetic properties of HS state. For LS configuration, metal-ligand bounds are stronger and shorter due to electron absence on *e*_*g*_, whereas for HS, the situation is opposite.

In the point charge model approximation of the ligand field theory, the energy gap between LS and HS states is determined in the following way
1

where *Ze* is the effective charge of the ligand, *R* is the metal-ligand distance, and *r*^4^ is the mean fourth power radius of the d-electron. This relation is fundamental because it shows that applied pressure may change directly the ligand filed splitting energy.

Thus, we have examined the thermal transition based on Ising-like spin-crossover Hamiltonian with applied hydrostatic pressure, where the stochastic nature of external field also have been taken into account.

## Methods

The complexity of structure of spin-crossover compounds lead to the appearance of a lot of models for describing the behavior of the main characteristics of these materials. We study the microscopic behavior of spin-crossover nanosystems based on the following general form of Ising-like Hamiltonian
2

where *s*_*i*,*j*_ are the pseudospin scalar variables which take the values ±1 for HS and LS states, respectively, *J* describes the site-to-site coupling, and *h*_0_ is an external field. For spin-crossover system, the external field (related to transition metal ion) is defined as follows:
3

Here, *Δ* is the ligand field splitting energy, describing the energy difference between HS and LS state *Δ*=*E*(HS)−*E*(LS) evaluated at zero temperature and atmospheric pressure, *k*_*B*_ is the Boltzmann constant, *T* is the temperature, and *g* is the degeneracy ratio between HS and LS states.

The Hamiltonian (2) describes in a convenient way the behavior of spin-crossover system where the control parameter is temperature. Great scientific interest also represents the case with applied hydrostatic pressure, that is one of more informative way to examine phase transition in spin-crossover materials. There are a lot of experimental works [11–13] where spin-crossover phenomena is studied by action of hydrostatic pressure where specific behavior of transition curves and therefore interesting practical properties (like piezochromism for pressure-induced hysteresis) were found.

The Hamiltonian of the system with hydrostatic pressure takes the form
4

where *p* is the external applied pressure, *ΔV* is the molecular volume change during transition between the spin states. The pressure action is introduced in the model taking into account its additive contribution to ligand field splitting energy. Since the applied pressure changes the molecular volume, the inter-molecular interaction is changed. Therefore, the high-order terms of interaction must be considered, but for simplicity, we use zero approach in molecular volume changes.

If we take into consideration the ligand field splitting energy in short time intervals, the system interaction with fluctuating external environment becomes relevant. This approach leads to breathing crystal field concept. The theoretical background and numerical simulations of the system with breathing crystal field are given in work [14]. Here, we describe the resulting Hamiltonian in framework of breathing crystal field concept with applied external pressure. The Hamiltonian takes the following form
5

where effective alternating field describing the result of interaction with environment is
6

The stochastic term *ξ*(*t*) represents the fluctuations of ligand field and for simplicity is considered as white Gaussian stochastic process with the following statistical characteristics


where *ε* is the strength of fluctuations. The stochastic behavior of spin-crossover system examined by macroscopic model in phenomenological approach is given in [15, 16].

We focus our study on the system described by the Hamiltonian (5) with the field (6) for various situations. We have to take under consideration that for such systems with alternating field (6) are impossible to obtain the information about their characteristics by analytical means, thus the way to study the systems with random component in external field may by carried out only by numerical methods. Here, we examined the spin-crossover system with free boundary conditions described by the Hamiltonian (5) by Monte Carlo (MC) simulations in the framework of Metropolis algorithm. The MC simulations were performed for the 2D Ising-like system on square lattice 40×40 and 1,000 MC steps. The main steps of Metropolis algorithm are (i) to fix the temperature, (ii) to fix the initial spin configuration, (iii) to find the system energy for initial configuration, (iv) to flip arbitrarily one spin from the system, (v) to find the system energy of new configuration, and (vi) to evaluate the transition probability of new configuration. Depending on the value of transition probability, this configuration may be accepted or rejected. If transition is accepted, the magnetization *m* of this configuration is found. This is one MC step. To have a good presentation of evolution of system order parameter, it is a need to carry out the numerical simulation for a sufficient number of MC steps, for which the system reaches the stationary regime. For each temperature, the resulted system magnetization *m* was found as average on MC steps *N*_MC_ from stationary regime of trajectory
7

where
8

Here, *L* is the lattice size of 2D Ising model.

The examined system was initialized with all spins up and high temperature enough, for which such spin configuration is maintained. If the temperature is decreasing, the spins are randomly flipped, and for certain temperature value, they become ordered in opposite state. In this way, we calculated the transition curves during cooling process. The transition probability of each spin for Metropolis algorithm is defined as follows:
9

Here, *ΔH*{*s*_*i*_} is the energy difference between two spin configurations (before and after transition of spin *s*_*i*_). In order to plot the heating branch of transition curve, the temperature is raised up to its initial value. From MC simulation by Metropolis algorithm with Hamiltonian (5), we cannot find directly the behavior of order parameter of spin-crossover system, i.e., the fraction of HS molecules *n*_HS_. Therefore first, we found the magnetization of Ising-like model as an average over MC steps from the trajectory that reaches the stationary regime. The relation between the magnetization of Ising model and order parameter of studied spin-crossover system, i.e., the fraction of HS molecules *n*_HS_ is the following:
10

The obtained numerical results based on these aproaches are presented in the next section.

## Results and discussion

**Figure 1:**
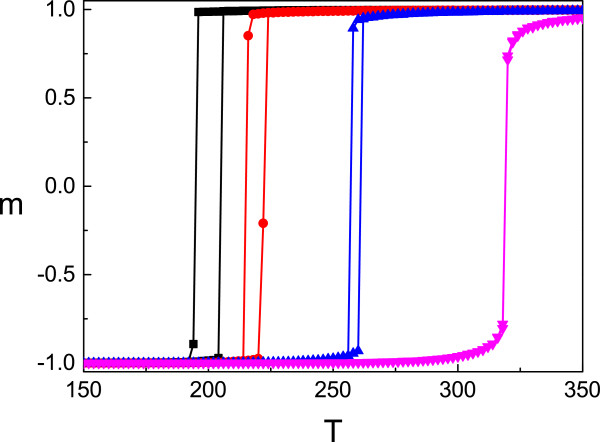
**The thermal transition curves for fixed values of external applied pressure.** The system’s parameters are the following: *g*=150, *Δ*=1,000 K, *L*=40. Here, the black curves correspond to the system without pressure; the red, blue, and pink ones are for the system with values *p* *Δ* *V*=100 K, *p* *Δ* *V*=300 K, and *p* *Δ* *V*=600 K, respectively.

The behavior of spin-crossover system under applied external pressure was examined basing on the transition curves obtained from Monte Carlo simulations of Hamiltonian (4). The corresponding results are reported in Figure 1. For simulations hereinafter, we chose the Boltzmann constant *k*_*B*_=1 (in Planck units) and the following system parameters: *g*=150, *Δ*=1,000 K, *L*=40, and 1,000 Monte Carlo steps per Kelvin degree. The product of the external pressure *p* and the change of molecular volume *ΔV* will measure in energy units of *T* similar to ref. [17]. Further, we postulated this product as pressure action because the volume change cannot be different in specific spin crossover material and this product may be changed only by variation of pressure *p*. The black curves in Figure 1 indicate pressureless system for spin-spin interaction *J*=145 K which is sufficient for thermal hysteresis. This value of spin-spin interaction is the same also for other curves from the figure. The red, blue, and pink curves are obtained for the pressure values *pΔV*=100 K, *pΔV*=300 K, and *pΔV*=600 K, respectively. Taking into account that for transition to HS state, the metal-ligand bounds increase and the action of external pressure prevents the bounds lengthening and increase the energy gap between the spin states; therefore, it leads to the shifting of the transition curves toward higher temperatures. The shifting of transition curves may be clearly observed from Figure 1 as the result of pressure increasing together with the vanishing of hysteresis width which takes place at the same time. If we turn to the standard units, it may be observed that the transition temperatures shift to the higher values with 20 K/kbar. This is in agreement with the experimental and theoretical data already reported in the paper [18]. Referring to the phase diagram, it is obvious that the influence of sufficient high external pressure moves the system on the phase diagram from the region of first-order phase transition to the region of second-order phase transition [12, 19, 20].

**Figure 2:**
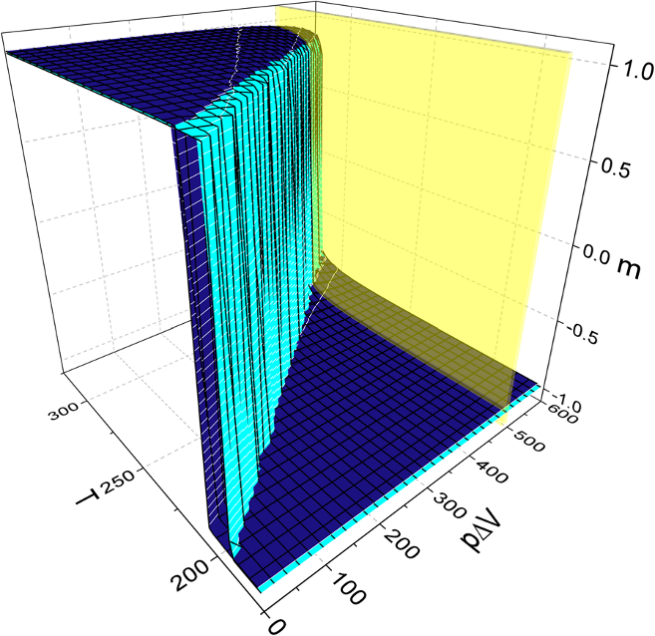
**The behavior of transition curves for simultaneous variation of temperature and** ***p*** ***Δ*** ***V*** **for interaction constant** ***J=145*** **K.**

The detailed characteristic of behavior of thermal transition curves on changing of applied pressure and temperature has been carried out, and the resulting three-dimensional plot has been built. It is presented in Figure 2. From this plot, we can analyze the changes of transition temperature with gradual increasing of the pressure. For the system with initial hysteresis, obtained for the interaction constant *J*=145 K, with pressure increasing, it may be distinguished from two different regions of system behavior that are divided in the figure by yellow plane. In the first region which is between *pΔV*=0 K and *pΔV*=500 K, the system shows hysteresis with decreasing width, and for the value of *pΔV*=500 K, it collapses. From this point begins the second region where system undergoes the second-order phase transition with shifting of transition curves toward higher temperatures if pressure is still increasing.

The vanishing of hysteresis width and shifts of the transition temperatures toward their higher values take place at the same time. For high values of interaction constant, the temperature shifts may be dominated upon hysteresis collapse, and therefore, the hysteretic behavior of the system at room temperatures may be observed. From a point of view of designing new IT devices based on spin-crossover compounds and their practical application, the hysteretic behavior at room temperature is a very important feature.

**Figure 3:**
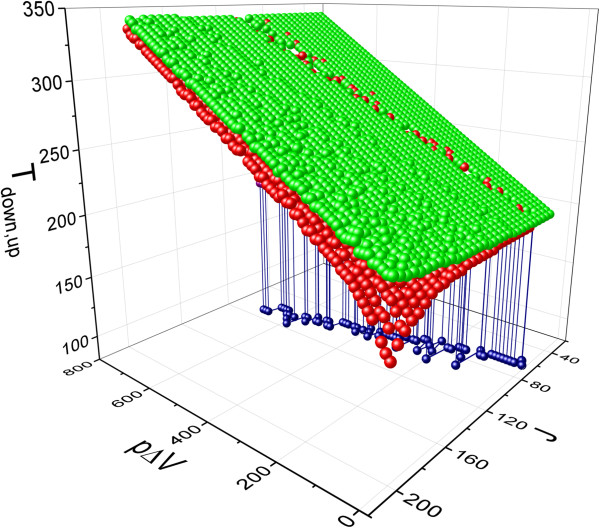
**The changes of transition temperatures during cooling and heating processes with increase of** ***p*** ***Δ*** ***V*** **and interaction constant** ***J*** **.**

In order to characterize the changes of hysteresis width for different interaction constant and pressure, we have built the 3D plot of transition temperatures during cooling and heating process *T*_down_ and *T*_up_. These temperatures are defined as equilibrium temperatures at which the fraction of HS molecules takes the value *n*_HS_=0.5. The obtained results are shown in Figure 3. Here, the red balls indicate the values of transition temperature during cooling *T*_down_, and green balls correspond to transition temperatures during heating *T*_up_. If it comes to hysteresis width, it can be judged from the difference between *T*_up_ and *T*_down_ for certain values of pressure and interaction constant. According to Figure 3, the hysteresis width decreases with a slight asymmetry with pressure increasing. The similar behavior is shown with decreasing interaction constant. In this plot, it is interesting to distinguish the behavior of transition temperature for which hysteresis collapses. The blue line in *J*−*pΔV* plane is the projection of edge of plane with equal values of *T*_down_ and *T*_up_. For the values of *J* larger than ones from the blue line, the second-order phase transition is observed. As we can see from Figure 3, the dependence between the values of *pΔV* and *J* for which hysteresis collapses is approximately linear.

**Figure 4:**
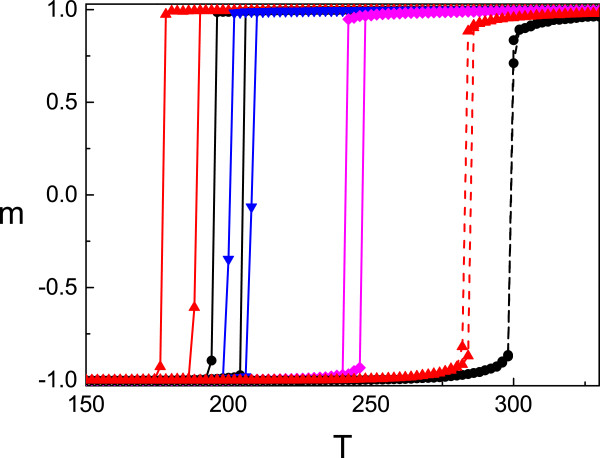
**The thermal hysteresis loop for system with random external action and applied pressure.** Here, the black solid curve is for the system without pressure and fluctuations and is the reference. The red, blue, and pink curves are for fixed fluctuations strength *ε*=100 for values *p* *Δ* *V*=0 K, *p* *Δ* *V*=100 K, and *p* *Δ* *V*=300 K, respectively. The dashed curves are for constant *p* *Δ* *V*=500 K which leads to the collapse of hysteresis for the system with *ε*=0 (black dashed curve). The red dashed curve is for *ε*=100 and *p* *Δ* *V*=500 and demonstrates the shift of threshold *p* *Δ* *V* value leading to hysteresis collapse. The interaction constant for all cases is *J*=145 K.

The more complete description of ligand field action on central transition metal ion requires to consider its instantaneous behavior where time variation of field becomes relevant. In the following study, we investigate the influence of fluctuating nature of ligand field splitting energy of spin-crossover system with applied pressure on thermal curves obtained from Monte Carlo simulations. Our starting point is Hamiltonian (5) with external field (6). The detailed investigation of transition curves for pressureless system with different statistical characteristics of stochastic term, i.e., space-time correlated fluctuations is carried out in paper [14]. Based on this approach, we consider the spin-crossover system under applied pressure. The resulting transition curves are shown in Figure 4. In the presented figure, the solid black transition curves correspond to the deterministic system with interaction constant *J*=145 K, described by the Hamiltonian (2). The red curves is for zero pressure and fluctuations strength *ε*=100. This strength is the same for other transition curves obtained for pressure *pΔV*=100 K (blue line) and *pΔV*=300 K (pink line). As it was expected, the presence of white fluctuations enlarges the hysteresis [14]. The pressure action on hysteresis loop for the system with fluctuations leads to its narrowing and, similar to the fluctuationless system, shifts the cooling and heating branches toward higher temperature. It is obvious that the action of pressure and fluctuations strength is opposite, and in such a way, it is expected that for the fluctuating system, the collapse of hysteresis takes place for higher pressure.

We are now turning to the comparison of the collapse of thermal hysteresis by raising the pressure in fluctuationless spin-crossover compound and in thepresence of fluctuations of ligand field. Numerical calculations for chosen interaction constant *J*=145 K for the system without fluctuations indicate that hysteresis collapses for *pΔV*=500 K, whereas in the presence of fluctuations with strength *ε*=100, the same transition curves which will coincide with presented ones (black dashed line in Figure 4) may be obtained for *pΔV*=580 K. If we plot the transition curves for the system with pressure *pΔV*=500 K and fluctuations strength *ε*=100 (red dashed line in Figure 4), the shift toward lower temperature is observed, which is the evidence of opposite to pressure action of fluctuations. Detailed analysis of thermal transition, essentially determined by intermolecular interaction *J*, which in more rigorous approach depends on the change of molecular volume, is however, beyond the scope of the present letter. It will also be a subject of future studies.

## Conclusions

We have presented an analysis of an Ising-like model with free boundary conditions that describes the spin-crossover nanomaterials under applied pressure. From Monte Carlo simulations based on Metropolis transition probabilities, the thermal transition curves for external field accounting its fast random variation in time were obtained. For spin-crossover nanosystems, the decreasing of hysteresis width by increasing of applied hydrostatic pressure is detected. It is found that the fluctuations of ligand field lead to opposite behavior related to applied pressure and widen the thermal hysteresis loop.

The applied pressure increases the energy gap between spin states and leads to the special feature of spin-crossover compounds that lies in the shifting of transition temperatures toward its room values. From a practical point of view, it is a very useful property for designing the nanosized devices based on spin-crossover complexes that may work at room temperature. It is shown that for hysteretic behavior at room temperature, which is an important feature for data storage devices, the spin-crossover systems with applied pressure requires high cooperative effects, i.e., the large interaction constant. It is concluded that the widening of hysteresis loop by fluctuations of ligand field in the presence of pressure may simplify the way to design memory and storage systems that need large hysteresis for their stable functioning.

The obtained results of numerical calculations are in agreement with the experiments [1, 2, 6, 11, 12, 18].
